# Awakening new sleep biology with machine learning

**DOI:** 10.1093/sleep/zsac284

**Published:** 2022-11-24

**Authors:** Mary Ann Hazuga, Struan F A Grant

**Affiliations:** Division of Human Genetics, The Children’s Hospital of Philadelphia, Philadelphia, PA, USA; Center for Spatial and Functional Genomics, The Children’s Hospital of Philadelphia, Philadelphia, PA, USA; Division of Human Genetics, The Children’s Hospital of Philadelphia, Philadelphia, PA, USA; Center for Spatial and Functional Genomics, The Children’s Hospital of Philadelphia, Philadelphia, PA, USA; Division of Diabetes and Endocrinology, The Children’s Hospital of Philadelphia, Philadelphia, PA, USA; Institute for Diabetes, Obesity, and Metabolism, Perelman School of Medicine at the University of Pennsylvania, Philadelphia, PA, USA; Department of Genetics, Perelman School of Medicine at the University of Pennsylvania, Philadelphia, PA, USA; Department of Pediatrics, Perelman School of Medicine at the University of Pennsylvania, Philadelphia, PA, USA

Shedding light on the genetics of sleep regulation has proven challenging despite the myriad of current approaches. The field has seen a degree of success in identifying variants that raise one’s susceptibility to sleep-related disorders [1–3]; however, such findings have only captured a small proportion of the predicted genetic contribution to such traits thus far. The main approach to study the genetics of sleep has been through the employment of genome-wide association studies (GWASs), which have implicated loci involved with conferring susceptibility to sleep dysregulation [4–6]. But GWAS outcomes do have key limitations in their interpretability, including the fact that the variants identified do not necessarily influence the expression of the nearest gene and thus the underlying effector genes remain to be elucidated [7, 8]. This has driven the development of tools for gene prioritization at loci associated with common complex traits. However, the methods applied to the study of sleep have been far from ideal due to assumptions that there are specific principal genes and pathways driving most of the phenotype [9–11]. The pathogenesis of sleep dysregulation and related traits are reported to be highly heterogeneous, driven across a variety of pathways and thus requiring advanced modeling to predict new connections that may not be immediately obvious. Lee et al. present a study integrating GWAS findings, known sleep genes and computational prediction models in an effort to build a more accurate picture of sleep regulation in humans [12].

This current study employs computational methods to infer more insight into genes and pathways impacting sleep regulation. Specifically, they trained machine learning models on a curated list of known sleep genes and from several large datasets representing a variety of studies. Machine learning has been a popular approach in the study of other complex diseases and has been employed previously to study both autism and Alzheimer’s disease [13, 14]. In the context of sleep, Lee et al. claim that using machine learning models can predict new genes and pathways based on similarity to what is already known about sleep genetics. The researchers mined the PubMed and Scopus literature resources to compile published sleep genes and to generate their list of 109 known targets. They then identified functional features that could separate sleep genes from non-sleep-related genes by leveraging the Jaccard similarity coefficient to quantify gene similarity and to use existing datasets to produce a profile for sleep genes. For the latter method, they started with over 7000 datasets and, following triaging based on measures of informativeness, were left with less than 100 that were determined to be informative for sleep [12].

There are several different machine learning models one can employ, each with their own set of advantages and disadvantages [15]. Lee et al. elected to utilize a biased learning method, given they were starting off with a set of positive labels, i.e. a list of known sleep genes [12]. The classifiers they chose to study within this category were as follows. Naive Bayes, which is based on conditional probability with assumed independence [15]. Support vector machines, which use a decision plane to separate data categories [15]. Decision trees and random forests (ensemble approach), which split data based on certain parameters [15]. And Neural networks (as well as ensemble neural networks), which optimize a path in an interconnected network [16]. All models were implemented with specific python packages, which are commonly used when building standardized machine learning approaches. For all the models, default parameters were used, with only a few minor exceptions [12]. With these parameters, the investigators found that the “random forest” model yielded the best performance based on their evaluation metrics, although in the discussion the authors do note that ensemble neural networks have higher sensitivity and could be useful in downstream analyses [12].

To evaluate their model, Lee et al. use *F*1 scores and area under the curve values [12]. *F*1 scores are a common metric for the evaluation of computational models and represent a balance between precision and recall scores [17]. Area under the curve is typically described as a metric for how well the model distinguishes between positive and negative classes [17, 18]. There are other metrics one can use to evaluate machine learning models [17], but these were selected in order to place emphasis on finding true positives in the data while also minimizing false positives that could lead to unnecessary and costly validation experiments.

Their model predicted >3000 genes related to sleep, with 95% of the known sleep genes also recalled by their model. Interestingly, they found that genes identified from human samples ranked higher than sleep genes identified from other organisms, despite the fact they were weighted equally during feature selection and model training. The authors used this phenomenon to note that their model can provide strong candidates for future studies in humans [12]. However, issues related to potential batch effects favoring human sleep genes probably warrant further investigation. Regardless, they used the top 495 genes from their model to carry out pathway enrichment analyses with the aim of identifying novel pathways related to sleep regulation.

The pathway enrichment analyses revealed highly established sleep regulation-related pathways, including circadian rhythm, G-protein, and neuron activity [4–6, 19–21]. Of these results, the investigators placed their subsequent focus on validating the implicated NF-κB pathway, which is a key process driving inflammation and immunity [22]. They used a mouse model setting where the transcription factor NF-κB was free to overexpress its target genes, which likely reflects chronic NF-κB activation. Ultimately, they found that this process led to sleep fragmentation, strongly suggesting that this pathway is connected to sleep regulation as they had predicted [12].

Beyond single pathway analyses, Lee et al. returned to in silico approaches to evaluate how the pathways they had identified were connected with respect to sleep regulation. A masked model approach [23] was used to identify effects on the prediction score for genes—where a given pathway category in turn was removed from the prediction model. They concluded that clock, NF-κB, and Ca^2+^ signaling were likely related in their impact on sleep regulation, as were G-protein, Phase 0—depolarization and ion channel signaling. Focusing on the first set of relationships, they identified several genes that were altered in their masked methods, noting that *TCF7L2* is impacted in both the masked-BioRhythm and masked-Inflammation models [12]. This indicates a possible link between both clock and inflammation pathways. Furthermore, the *TCF7L2* locus has been strongly implicated in the pathogenesis of type 2 diabetes [24], a trait strongly correlated with sleep dysregulation—so this observed relationship may warrant further investigation.

Overall, Lee et al. present an intriguing method for prioritizing new genes and pathways related to complex disease that may have not been detected by previous methods. They do note that their approach is limited in that it cannot address causal relationships in sleep on its own, rather only provide predictions [12]. This is true of many computational methods, as functional validation is important ultimately to identify if the predicted relationship is biologically accurate [8]. It is also limited by the amount of related data available for sleep. Despite these drawbacks, this method, along with using machine learning in general, can help guide new functional experiments on genes that were previously uncharacterized in the context of a given complex disease, creating the opportunity to uncover new and interesting connections while also reducing time and money spent on experimentation.
